# Fracture and compaction of andesite in a volcanic edifice

**DOI:** 10.1007/s00445-015-0938-7

**Published:** 2015-06-03

**Authors:** M. J. Heap, J. I. Farquharson, P. Baud, Y. Lavallée, T. Reuschlé

**Affiliations:** Équipe de Géophysique Expérimentale, Institut de Physique de Globe de Strasbourg (UMR 7516 CNRS, Université de Strasbourg/EOST), 5 rue René Descartes, 67084 Strasbourg cedex, France; Earth, Ocean and Ecological Sciences, University of Liverpool, Liverpool, L69 3GP UK

**Keywords:** Outgassing, Volcán de Colima, Brittle, Inelastic compaction, Pore collapse, Shear fracture, Edifice stability, Permeability, Stratovolcano

## Abstract

The failure mode of lava—dilatant or compactant—depends on the physical attributes of the lava, primarily the porosity and pore size, and the conditions under which it deforms. The failure mode for edifice host rock has attendant implications for the structural stability of the edifice and the efficiency of the sidewall outgassing of the volcanic conduit. In this contribution, we present a systematic experimental study on the failure mode of edifice-forming andesitic rocks (porosity from 7 to 25 %) from Volcán de Colima, Mexico. The experiments show that, at shallow depths (<1 km), both low- and high-porosity lavas dilate and fail by shear fracturing. However, deeper in the edifice (>1 km), while low-porosity (<10 %) lava remains dilatant, the failure of high-porosity lava is compactant and driven by cataclastic pore collapse. Although inelastic compaction is typically characterised by the absence of strain localisation, we observe compactive localisation features in our porous andesite lavas manifest as subplanar surfaces of collapsed pores. In terms of volcano stability, faulting in the upper edifice could destabilise the volcano, leading to an increased risk of flank or large-scale dome collapse, while compactant deformation deeper in the edifice may emerge as a viable mechanism driving volcano subsidence, spreading and destabilisation. The failure mode influences the evolution of rock physical properties: permeability measurements demonstrate that a throughgoing tensile fracture increases sample permeability (i.e. equivalent permeability) by about a factor of two, and that inelastic compaction to an axial strain of 4.5 % reduces sample permeability by an order of magnitude. The implication of these data is that sidewall outgassing may therefore be efficient in the shallow edifice, where rock can fracture, but may be impeded deeper in the edifice due to compaction. The explosive potential of a volcano may therefore be subject to increase over time if the progressive compaction and permeability reduction in the lower edifice cannot be offset by the formation of permeable fracture pathways in the upper edifice. The mode of failure of the edifice host rock is therefore likely to be an important factor controlling lateral outgassing and thus eruption style (effusive versus explosive) at stratovolcanoes.

## Introduction

Volcanic edifices, products of the accumulation of successive lava and volcaniclastic deposits and endogenous growth (Borgia and Linneman 1990; Kaneko 2002; Biggs et al. 2010), play a central role in governing volcanic hazards (Voight 2000). First, the structural stability of the edifice, and therefore its susceptibility to catastrophic collapse, depends on the integrity of this rapidly emplaced mélange of coherent lava flows and poorly consolidated volcaniclastic deposits (e.g. Gudmundsson 2011). Second, the ease with which exsolving magma can outgas into the country rock (e.g. Jaupart 1998; Collinson and Neuberg 2012), a factor dictating the explosivity of the volcano, relies on the physical state (porosity, permeability) of the edifice host rocks (e.g. Eichelberger et al. 1986; Woods and Koyaguchi 1994; Mueller et al. 2008; Nguyen et al. 2014; Castro et al. 2014; Okumura and Sasaki 2014; Gaunt et al. 2014; Farquharson et al. 2015). Throughout edifice construction, edifice rocks are subject to a multitude of local and regional stresses that persistently alter their physical state, challenging edifice stability and influencing lateral outgassing; for example, local stress fields can rapidly change due to dyke propagation, regional stresses exist in the form of tectonic stresses, and lithostatic stresses build as effusive and explosive products that accumulate over time (e.g. Roman et al. 2004; Gerst and Savage 2004; Gudmundsson 2006). As a result, during the life cycle of a volcano, the initially steep conical structure evolves into a more dispersed and degraded landform (van Wyk de Vries and Borgia 1996; Borgia et al. 2000). Ultimately, this increasingly unstable structure can collapse, evidence of which is exposed in the geological record as sector collapse scars, amphitheatres, craters and calderas (e.g. Guest et al. 1984; Stoopes and Sheridan 1992; Hall et al. 1999; Tibaldi 2001). It follows that the mechanical response of the rocks that comprise the edifice to regional and local stresses must represent a fundamental factor in the progressive destabilisation of a volcano and the evolution of outgassing efficiency and thus explosivity.

When exposed to a differential stress, porous rock reacts in one of two ways. The porosity within the rock (a combination of microcracks and pores) will either increase (dilation) or decrease (compaction). The operative micromechanical process, dilatational microcracking or compactive pore collapse/grain crushing, dictates the response of the rock to an applied stress and is dependent on both the initial physical properties of the rock (e.g. porosity, pore size) and the conditions (e.g. pressure, temperature, pore fluid) under which the rock deforms (see the review by Wong and Baud 2012 and references therein). At low confining pressures (shallow depths), both low- and high-porosity rocks will dilate resulting in a dilatant mode of failure, such as axial splitting (at very low confining pressures or depths) or shear failure (e.g. Paterson and Wong 2005). However, as confining pressure (depth) increases, while low-porosity rock will continue to form shear fractures, high-porosity rock will undergo shear-enhanced compaction driven by cataclastic pore collapse and grain crushing (Wong and Baud 2012).

Importantly, the mode of failure will severely impact the evolution of rock physical properties. Laboratory experiments have shown that shear fracturing (and associated dilatancy) is synonymous with an increase in porosity (Read et al. 1995) and a decrease in elastic wave velocity (Ayling et al. 1995). Some experimental data, however, suggest the impact of fracturing on permeability may depend on the initial porosity of the rock. While dilation and the formation of a macroscopic shear fracture (e.g. Zoback and Byerlee 1975; Mitchell and Faulkner 2008) and tensile (extension) fractures (Nara et al. 2011) have been shown to increase the permeability of low-porosity rock by many orders of magnitude, experiments on high-porosity (>15 %) sandstones have shown that shear fractures can decrease permeability (Zhu et al. 1997a; Ngwenya et al. 2003). Indeed, some field studies on large faults in porous rocks have shown that permeability decreases as the fault is approached (Shipton et al. 2002; Farrell et al. 2014). Similar studies on large faults in low-porosity rock attest to a significant increase in permeability within the adjacent damage zone (Mitchell and Faulkner 2012), although the low permeability of the fault core can impart a permeability anisotropy (Faulkner and Rutter 2001; Wibberley and Shimamoto 2003).

By contrast, inelastic compaction will serve to increase elastic wave velocity (Fortin et al. 2005), decrease porosity (Wong and Baud 2012) and, in all cases, decrease permeability (David et al. 1994; Zhu et al. 1997b; Fortin et al. 2005; Baud et al. 2012). The failure mode also influences the output of acoustic emissions (AE, typically used as a proxy for microcracking) during deformation (Wong et al. 1997). An understanding of the mechanical behaviour and failure modes, and their impact on rock physical properties, of edifice-forming volcanic rocks is therefore of upmost importance. For example, the efficiency of lateral outgassing through the country rock (e.g. Jaupart 1998) is likely aided by a dilatant failure mode and hindered by a compactant failure mode.

Laboratory studies on the mechanical behaviour and failure modes of rock have been biased towards sedimentary rocks (Wong and Baud 2012). Studies on volcanic rocks—rocks with a greater microstructural complexity—are few (e.g. Kennedy et al. 2009; Zhu et al. 2011; Loaiza et al. 2012; Adelinet et al. 2013; Heap et al. 2014a, 2015), but have highlighted that volcanic rock can switch from dilatant to compactive modes of failure as effective pressure (i.e. depth) is increased. High-porosity tuffs (30–50 %) have been shown to switch to inelastic compaction at very low effective pressures (Peff = 5–10 MPa; Peff = Pc − *α*Pp, where Pc and Pp are the confining pressure and pore pressure, respectively, and poroelastic constant *α* is assumed to be 1), corresponding to depths of a couple of hundred metres (Zhu et al. 2011; Heap et al. 2014a, 2015). Studies on porous extrusive rocks have shown that inelastic compaction is encountered at much higher effective pressures. An aphanitic basalt from Reykjanes (Iceland) containing a porosity of 8 % switched to compactive behaviour at an effective pressure of 75 MPa (Adelinet et al. 2013), while an aphanitic trachyandesite from the Açores (Portugal) with a porosity of 18 % was compactant at 90 MPa (Loaiza et al. 2012), pressures corresponding to depths greater than 3 km. Kennedy et al. (2009) showed that low-porosity (8 %) dacite from Mount St. Helens (USA) exhibited shear faulting up to effective pressures of 75 MPa, while the deformation of high-porosity (20–24 %) dacite from Augustine volcano (USA) was driven by distributed cataclastic flow at pressures of 25 MPa and higher. Despite these studies, the paucity of experimental data on the mechanical behaviour and failure modes of volcanic rock inhibits our understanding, a key element to interpret the evolution of edifice stability and sidewall outgassing. For instance, the rocks comprising a volcanic edifice are known to be variably porous (e.g. Melnik and Sparks 2002; Kueppers et al. 2005; Lavallée et al. 2012; Farquharson et al. 2015). However, little is known about the influence of porosity on the failure mode of representative edifice-forming rocks. To better understand the deformation of edifice-forming rock, we conducted a systematic experimental study on the mechanical behaviour and failure mode of a suite of edifice-forming andesites containing different porosities (7 to 25 %), deformed under volcanogically relevant pressures (corresponding to depths from a couple of hundred metres to about 3 km).

## Case study, materials and methods

### Case study: Volcán de Colima

**Fig. 1:**
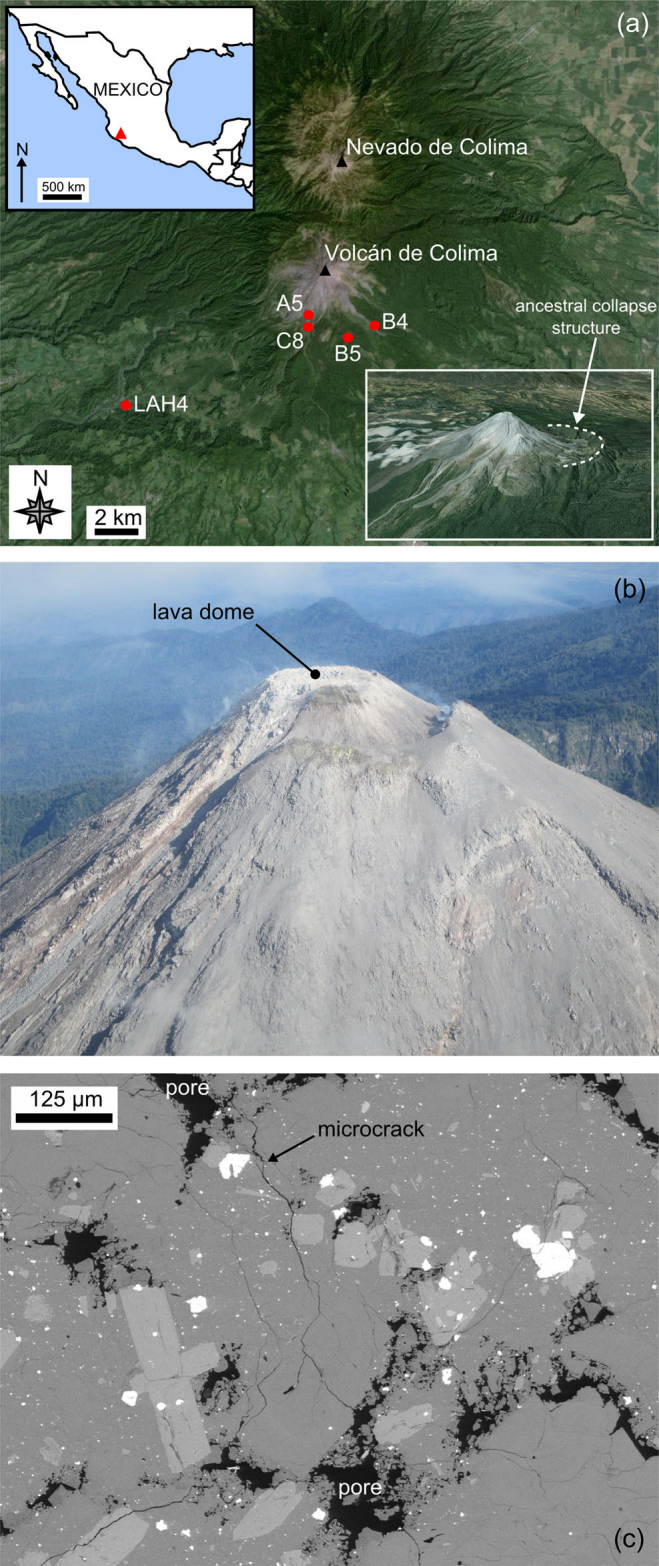
**a** Google Earth^TM^ map showing the locations of the sampling sites with respect to Volcán de Colima and Nevada de Colima. *Insets* show a map of Mexico (the *red triangle* corresponds to the position of Volcán de Colima) and a Google Earth^TM^ image of Volcán de Colima showing the ancestral collapse structure (*dashed white line*). **b** Aerial photograph of the dome at Volcán de Colima (May 2014; photo credit: M. Heap). **c** Scanning electron microscope image showing the porosity network with a sample of andesite (B5) from Volcán de Colima. The microstructural elements are identified on the figure

For the purpose of this study, we selected edifice-forming andesitic rocks from Volcán de Colima (Trans-Mexican Volcanic Belt, Mexico, 19° 30′ N, 103° 37′ W, Fig. 1). Volcán de Colima was specifically chosen for this study as it is an active and frequently collapsing andesitic stratovolcano, with a construction and eruption history comparable to other active andesitic stratovolcanoes observed worldwide, such as Merapi (Indonesia), Santa María (Guatemala), Tungurahua (Ecuador) and Ruapehu (New Zealand). The volcanic complex comprises the active Fuego de Colima, constructed in the amphitheatre of an earlier collapse structure, and the older and extinct edifice of Nevado de Colima (Fig. 1). The most recent collapse event (2550 bp) was the last of at least five major collapses during the last 18,500 years (Stoopes and Sheridan 1992; Cortés et al. 2010). More recent activity has been characterised by lava effusion and Vulcanian explosions sandwiched between Plinian and sub-Plinian eruptions; these major explosive eruptions are thought to occur about every 100 years (Luhr 2002). Present day eruptive activity is extensively monitored through seismicity (Arámbula-Mendoza et al. 2011; Lamb et al. 2014; Lesage et al. 2014), gas geochemistry (Taran et al. 2002; Varley and Taran 2003), thermal infrared imaging (Hutchinson et al. 2013; Stevenson and Varley 2008; Webb et al. 2014), rockfall (Mueller et al. 2013) and deformation (Zobin et al. 2002). Between November 1998 and June 2011, there were five episodes of dome growth. Slow effusion and dome growth occurred in 2001–2003 and 2007–2011, interrupted by much faster episodes in 1998–1999, 2004 and 2005 (Varley et al. 2010). Explosive activity during this time was characterised by small gas-and-ash events and larger dome-disrupting Vulcanian events. The most intense period of activity provided at least 30 explosions, generating pyroclastic flows that reached distances as far as 5.4 km from the active vent (Varley et al. 2010). The most recent eruptive sequence, which started in January 2013, has involved dome growth and lava extrusion punctuated by pyroclastic density currents and Vulcanian explosions. Frequent explosive events were ongoing at the time of writing (i.e. May 2015).

### Experimental materials

We selected four andesitic lava blocks (typically 30 × 30 × 30 cm) to represent the variation in porosity typically seen within the materials forming the edifice at Volcán de Colima. A recent field-based study at Volcán de Colima (Farquharson et al. 2015) revealed the porosity of the eruptive products to be between 2 and 75 % (based on 542 hand samples). Using the method of Bernard et al. (2015), a weighted abundance analysis of these data shows that the predominant porosity class at Volcán de Colima is between 10 and 25 %. Using a similar field density technique, Mueller et al. (2011) found an average porosity of 16.4 % (based on 299 hand samples; see also Lavallée et al. 2012) and Lavallée et al. (2015) found that the average porosity of 2635 hand samples to be about 20 % (porosity ranged from 8 to 40 %). The range of porosities studied herein (from 7 to 25 %) is therefore representative of the rocks most frequently observed in the field.

The first block, A5, is from the 1998–1999 lava flow in the Cordoban ravine and contains a connected porosity of about 11 %. B5 is from an older lava flow of unknown age and contains a connected porosity of around 8 %. We note that B5 displays a certain degree of high-temperature alteration, as evidenced by the presence of vapour-deposited cristobalite within the pores (Fig. 1c; see Horwell et al. 2013 and Schipper et al. 2015). Block C8 was taken from the 1998–1999 blow-and-ash flow in the San Antonio ravine and contains a connected porosity of about 17 %. Finally, LAH4 is a block of unknown age collected from a lahar deposit on the west flank of the volcano (in the El Zarco river bed near La Becerrera); LAH4 contains a connected porosity of approximately 25 %. The locations of the collection sites are indicated in Fig. 1a. Using the classification scheme of Farquharson et al. (2015), B5 can be classified as “altered lava” and A5, C8 and LAH4 as “lava”. All of the andesite blocks contain a dual porosity: a combination of microcracks and pores (Fig. 1c, Heap et al. 2014b). In detail, the andesites are pervasively microcracked (containing average microcrack densities between 35 and 45 mm^−1^) and contain high pore number densities (between 3.3 and 8.1 mm^−2^) and wide pore size distributions (the pore diameters range between about 0.02 and 2.0 mm; Heap et al. 2014b). The andesites have a porphyritic texture containing a microlitic groundmass (59–68 %) containing commonly microcracked phenocrysts (<1.5 mm in diameter) of plagioclase (13–25 %), clinopyroxene (3–4 %) and orthopyroxene (2–4 %). All of the andesites contain between 58 and 61 wt% silica (Heap et al. 2014b), compositionally representative of recently erupted materials from Volcán de Colima (Luhr 2002; Savov et al. 2008). Cylindrical core samples, cored in the same orientation to a diameter of 20 mm and precision-ground to a nominal length of 40 mm, were prepared from each of the blocks. The connected water porosities of the samples were measured using the triple weight water-saturation (distilled water) method.

### Experimental methods

All experiments were performed at the Géophysique Expérimentale laboratory at the Institut de Physique du Globe de Strasbourg. Uniaxial compressive strength (UCS; *σ*_1_ > *σ*_2_ = *σ*_3_ = 0) experiments were performed on water-saturated samples of each andesite at a constant strain rate of 10^−5^ s^−1^ until failure. During uniaxial compression, axial stress was measured using a load cell and axial strain via a displacement transducer. The water-saturated samples were deformed inside a bath of distilled water. Triaxial deformation experiments were performed using a conventional triaxial apparatus (*σ*_1_ > *σ*_2_ = *σ*_3_) on water-saturated samples at a constant strain rate of 10^−5^ s^−1^. Our chosen strain rate is the standard for rock deformation experiments in compression, allowing our data to be compared with the wealth of pre-existing data (see review by Wong and Baud 2012). All triaxial experiments were performed under drained conditions. The pore fluid pressure was kept at a constant 10 MPa, and we ran experiments at confining pressures between 15 and 80 MPa (i.e. Peffs between 5 and 70 MPa), equivalent to depths between a couple of a hundred metres to about 3 km. For the purpose of this study, we assume a simple effective pressure (Peff) law such that Peff = Pc − *α*Pp, where poroelastic constant *α* is assumed to be 1. Prior to deformation, the samples were left at the target effective pressure for at least 12 h to ensure microstructural equilibrium. During experimentation, we measured axial stress via a load cell and axial strain using a displacement transducer located on the top piston. Porosity change was measured using a pore pressure intensifier/volumometer and the output of acoustic emissions (AEs) and AE energy (the area under the received AE waveform) using a piezoelectric crystal attached to the top piston. Hydrostatic experiments—during which the confining pressure acting on a sample is increased while maintaining a constant pore fluid pressure—were also performed on a sample of each andesite. No differential stress is imposed on the sample during these experiments (i.e. *σ*_1_ = *σ*_2_ = *σ*_3_). To ensure microstructural equilibration, the samples were first left for at least 12 h under a confining pressure of 12 MPa and a pore pressure of 10 MPa. The confining pressure was increased at a servo-controlled rate of 0.003 MPa s^−1^, and the porosity change was monitored during the experiments using a pore pressure intensifier/volumometer. Details of the triaxial experimental apparatus can be found in a previous contribution (Heap et al. 2014a). All of the experiments reported in this study were performed at room temperature. The focus of this study is to characterise the mechanical behaviour of edifice-forming andesites, which have long since cooled below the glass transition temperature (Tg) of their melt phase (**~**740 °C, Lavallée et al. 2012). While we are confident that viscous deformation will only occur within edifice rock in contact with a heat source (e.g. a dyke), we are aware that elevated temperatures may encourage subcritical crack growth (Brantut et al. 2013), although we note that increasing the temperature from room temperature to 75 °C did not significantly influence the deformation rate during a long-term triaxial experiment on a basalt from Mt Etna (Brantut et al. 2013). At the strain rates studied herein, we do not expect a temperature-induced change in failure mode at temperatures below Tg, exemplified by the brittle and dilatant behaviour of basalt and crystallised dacite samples deformed triaxially at high temperature (up to 900 °C; Smith et al. 2011; Violay et al. 2015). In this study, we adopt the convention that compressive stresses and strains are positive. An experimental summary, containing all of the data collected for this study, is given as Table 1.

**Table 1:** Experimental summary of the 39 experiments performed for this study. All experiments were performed at the Géophysique Expérimentale laboratory at the Institut de Physique du Globe de Strasbourg. C* - onset of shear-enhanced compaction; P - effective mean stress; P* - onset of lithostatic inelastic compaction; N/A - not available (sample was too strong to break in our experimental setup under these pressure conditions)

Block	Sample	Connected porosity (%)	Confining pressure (MPa)	Pore pressure (MPa)	Effective pressure (MPa)	Peak differential stress (MPa)	C* (MPa)	*P* (MPa)	P* (MPa)	Notes
B5	4_s1	7.9	0	0 (wet)	0	81.1	–	27.0	–	
B5	8	7.3	15	10	5	136.0	–	50.3	–	
B5	7	7.4	20	10	10	184.9	–	71.6	–	
B5	10	7.9	40	10	30	270.7	–	120.2	–	
B5	11	7.5	60	10	50	281.4	–	143.8	–	
B5	4	7.7	80	10	70	N/A	–	–	–	
B5	2	7.6	Hydro	10	Hydro	–	–	–	N/A	
B5	3	7.6	Hydro	10	Hydro	–	–	–	N/A	
A5	7	12.3	0	0 (wet)	0	64.8	–	21.6	–	
A5	17	9.3	15	10	5	128.7	–	47.9	–	
A5	10_s1	11.2	20	10	10	164.2	–	64.7	–	
A5	4_s1	11.7	40	10	30	209.1	–	99.7	–	
A5	14_s1	10.6	60	10	50	261.7	–	137.2	–	
A5	4	11.2	80	10	70	–	290.1	166.7	–	
A5	20	9.8	Hydro	10	Hydro	–	–	–	N/A	
C8	5_s1	17.6	0	0 (wet)	0	17.5	–	5.8	–	
C8	16	16.2	15	10	5	74.1	–	27.7	–	Microstructure
C8	4_s1	17.9	20	10	10	62.3	–	30.8	–	
C8	19	19.4	40	10	30	–	43.4	44.5	–	
C8	23	18.5	40	10	30	–	48.7	46.2		
C8	20	17.6	60	10	50	–	45.3	65.1	–	
C8	8	15.5	60	10	50	–	103.5	84.5	–	C*′ (20 % strain)
C8	21	16.5	60	10	50	–	60	70	–	Microstructure; 1.5 % strain
C8	5	16.3	60	10	50	–	78.3	76.1	–	Microstructure; 3 % strain
C8	4	16.4	60	10	50	–	60.3	70.1	–	Microstructure; 6 % strain
C8	26	16.7	60	10	50	–	59.5	69.8	–	Permeability, 1.5 % strain
C8	25	17.2	60	10	50	–	49.4	66.5	–	Permeability, 4.5 % strain
C8	22	19.0	80	10	70	–	26.5	78.8	–	
C8	6	16.7	Hydro	10	Hydro	–	–	–	126.0	
C8	7	16.7	Hydro	10	Hydro	–	–	–	151.7	Microstructure
C8	i	17.2	0	0	0	fractured in tension	–	–	–	Permeability
C8	ii	18.1	0	0	0	fractured in tension	–	–	–	Permeability
LAH4	7	23.8	0	0 (wet)	0	31.3	–	10.4	–	
LAH4	1	24.1	20	10	10	69.5	–	33.2	–	
LAH4	2	24.0	40	10	30	–	92.5	60.8	–	
LAH4	4	24.2	60	10	50	–	72.9	74.3	–	
LAH4	6	24.5	80	10	70	–	56.4	88.8	–	
LAH4	8	23.8	Hydro	10	Hydro	–	–	–	160.6	
LAH4	9	23.8	Hydro	10	Hydro	–	–	–	150.0	

## Failure mode: dilatant or compactant?

The mechanical behaviour of rock is often classified as brittle or ductile (Rutter 1986; Evans et al. 1990; Paterson and Wong 2005; Wong and Baud 2012). Shear fracturing, a product of the coalescence of (predominately tensile) microcracks, is described as a brittle mode of failure. Ductile behaviour, however, defined simply as the capacity of a material to deform to a substantial strain without the tendency to localise the flow into faults (Rutter 1986), can be the result of a variety of microstructural deformation mechanisms, including microcracking (in the case of cataclastic flow); the description of ductility holds no mechanistic connotation (Rutter 1986). However, due to instances of compaction localisation (e.g. Baud et al. 2004), and because ductile behaviour can be driven by microcracking (i.e. “brittle” on the microscale), we have simplified our classification of the failure mode of rock in this manuscript to “dilatant” and “compactant”.

**Fig. 2:**
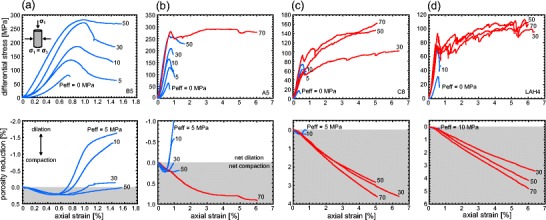
Mechanical data. Stress-strain curves and porosity reduction curves for andesitic lava from Volcán de Colima: **a** B5, **b** A5, **c** C8 and **d** LAH4. The effective pressure (*Peff*) of the experiment is shown next to each curve. Dilatant curves are shown in *blue* and compactant curves are shown in *red*. Net compaction in the graphs of porosity reduction is highlighted in *grey*, net dilation in *white*

**Fig. 3:**
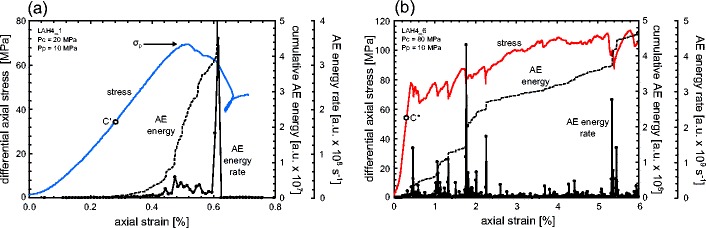
Acoustic emission characteristics. Cumulative acoustic emission energy (*AE*) and AE energy rate (AE energy is given in arbitrary units, a.u.) during **a** a dilatant constant strain rate experiment (Peff = 10 MPa) and **b** a compactant constant strain rate experiment (Peff = 70 MPa) on porous andesite. The experiments shown here were performed on samples of LAH4 (the same experiments presented in Fig. 2d). The positions of the onset of dilatational microcracking (*C*′) and the peak stress (*σ*_p_) are indicated in panel **a**, and the position of the onset of shear-enhanced compaction (C*) is indicated in panel **b**

Stress-strain curves and porosity reduction-strain curves for each of the andesite lavas, for different effective pressures (from 0 to 70 MPa or depths from 0 m to 3.2 km), are shown in Fig. 2. Dilatant behaviour (blue curves) is characterised by strain softening and large stress drops, typically associated with shear fracture formation (Fig. 2). The convex shape of the initial portion of the stress-strain curves (e.g. Fig. 2a) is typically attributed to the closure of microcracks aligned sub-perpendicular to the loading direction. Indeed, the initial portion of the porosity reduction curves shows that the lava is compacting (e.g. Fig. 2a). The lavas then enter an elastic deformation stage where the stress-strain curve is quasi-linear, followed by a stage where the curves are concave. At the beginning of this latter stage, microcracks nucleate and grow (inelastic deformation). The onset of dilatancy, termed *C*′ (Wong et al. 1997), is best observed using porosity change measurements (see discussion below) but can usually be observed as the start of an acceleration in AE activity (Fig. 3a), used as a proxy for the nucleation and growth of microcracks (e.g. Lockner 1993). The onset of dilatant microcracking can be observed as a reduction in the rate of porosity decrease in the porosity reduction curves (e.g. Fig. 2a; Wong et al. 1997), and eventually, as the rate of microcracking accelerates, the lava switches from compaction-dominated behaviour to dilation-dominated behaviour. The rate of microcracking, monitored by the output of AE (Fig. 3a), continues to accelerate up to the peak stress (*σ*_p_). Following the peak stress, there is a strain softening phase before the lava succumbs to macroscopic failure, marked by a large stress drop and a rapid acceleration in AE activity (Fig. 3a). The stress-strain curves for the lavas are typical of those for rock in compression (e.g. Hoek and Bieniawski 1965; Brace et al. 1966; Scholz 1968). We note that, for the dilatant lavas, the peak stress and the strain-at-failure increases, and the magnitude of the stress drop decreases, with increasing effective pressure (see also Paterson and Wong 2005). We also highlight that the porosity reduction curves show that samples deformed at higher effective pressures show less net dilation (e.g. Fig. 2a). Visual inspection of the deformed samples confirmed that the samples contained localised shear fractures typically orientated at about 30° to the maximum principal stress.

Compactant behaviour (red curves) of the andesitic lavas is characterised by the lack of significant strain softening, strain hardening (in some cases) and many small stress drops (of a couple of MPa) (Fig. 2). Similar to the dilatant curves, the compactant curves contain an initial convex portion, associated with the closure of microcracks (the porosity reduction curves show that the lava is compacting; e.g. Fig. 2c, d) and an elastic deformation stage where the stress-strain curve is approximately linear. However, unlike the dilatant curves, there is no switch to dilation dominance. At a critical stress state, termed the onset of shear-enhanced compaction or C* (Wong et al. 1997), the rate of compaction increases (e.g. Fig. 2c, d). As for *C*′, C* is best observed using porosity change measurements (see discussion below) but also usually marks the position of the onset of significant AE activity (Fig. 3b) whereat the lava begins to deform inelastically. We also note the presence of many small stress drops that are contemporaneous with sudden and temporary increases in the rate of AE output (Fig. 3b); such stress drops and AE bursts have previously been attributed to compaction localisation in porous rock (Baud et al. 2004; this is discussed further in the “Operative micromechanical processes” section). Unlike failure in the dilatant regime, the differential stress required for the onset of shear-enhanced compaction decreases with increasing effective pressure. Our experiments highlight that the rate of compaction increases as the effective pressure increases (e.g. Fig. 2d); for example, at 6 % axial strain, LAH4 had lost about 3 and 5 % porosity at effective pressures of 30 and 70 MPa, respectively.

The transition between dilatant and compactant behaviour was observed at effective pressures of 30 MPa and above (equivalent to depths greater than about 1.6 km) for the higher porosity lavas (C8 and LAH4), while the failure mode of the samples from the blocks containing the lowest porosities (B5 and A5) remained dilatant up to 50 MPa. Above 50 MPa (depth ~2.4 km), A5 switched from a dilatant to a compactive failure mode (B5 was too strong to break in our triaxial press at a Peff of 70 MPa).

**Fig. 4:**
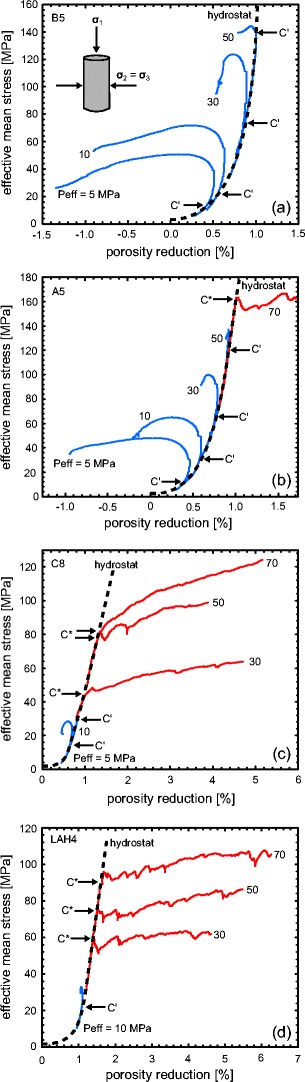
Plots of porosity reduction against effective mean stress for andesitic lava from Volcán de Colima (the same experiments presented in Fig. 2): **a** B5, **b** A5, **c** C8 and **d** LAH4. The effective pressure (Peff) of the experiment is shown next to each curve. Dilatant curves are shown in *blue* and compactant curves are shown in *red*. The positions of the onset of dilatational microcracking (*C*′) and the onset of shear-enhanced compaction (C*) are indicated where appropriate. The hydrostatic curves (“hydrostats”) are given as *black dashed lines*

Additional insights into the mechanical behaviour of the andesites can be gleaned by plotting the porosity reduction versus the effective mean stress (*P*), where *P* = ((*σ*1 + 2*σ*3)/3) − Pp. Such curves highlight the difference between hydrostatic (*σ*_1_ = *σ*_2_ = *σ*_3_) and shear stresses (*σ*_1_ > *σ*_2_ = *σ*_3_) on the evolution of porosity (Fig. 4). In the hydrostatic case, the onset of inelastic compaction is termed P* (Wong et al. 1997; Fig. 4). P* was attained for the two most porous samples (C8 and LAH4; see Table 1), but A5 and B5 contain porosities too low to observe P* in our experimental setup. We note that, in all cases, an increase in hydrostatic stress resulted in a decrease in porosity. Prior to P*, this is attributed to the elastic closure of porosity as pressure is increased; the acceleration in porosity loss following P* is attributed to inelastic compaction (Wong et al. 1997). Any deviation from the hydrostatic curve (or “hydrostat”) during a constant strain rate triaxial experiment must therefore be the consequence of differential stress on the porosity evolution. A dilatant mode of failure is characterised by a deviation to the left (porosity increase), marked by *C*′, and a compactant mode of failure by a deviation to the right (porosity decrease), marked by C* (see Wong et al. 1997).

## Constructing failure envelopes for porous andesite

**Fig. 5:**
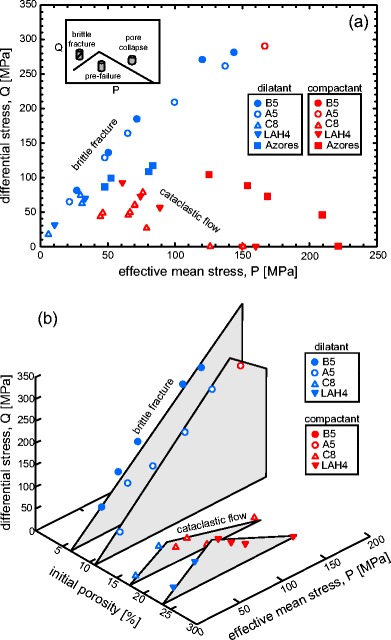
Failure envelopes for andesitic lava from Volcán de Colima. **a** The experimental data plotted on differential stress *Q* at failure versus effective mean stress *P*. **b** A 3D plot of differential stress at failure and effective mean stress plotted alongside the initial connected porosity. Dilatant experiments are shown as *blue symbols* and compactant experiments as *red symbols*. Data for Açores trachyandesite (*squares*) from Loaiza et al. (2012) is also presented in panel **a**

The data of this study can be used to map failure envelopes for andesite lava containing different porosities (Fig. 5). In the dilatant regime, the peak stress maps the dilatant failure envelope on a plot of differential stress (*Q*) versus effective mean stress. In the compactant regime, it is the stress at the onset of shear-enhanced compaction C* that delineates the compactive yield envelope. The positions of P*, lithostatic inelastic compaction, plot along the *x*-axis (*Q* = 0 MPa). The lava has failed (or yielded) if the stress state plots outside the failure envelope (shear fracture on the left and inelastic compaction on the right; see inset in Fig. 5a). It follows that stronger rocks will therefore be intact over a much larger *P*-*Q* space (i.e. the failure envelope will have a larger amplitude).

The complete failure envelopes are only available for the most porous lavas (C8 and LAH4); the low-porosity lavas (A5 and B5) were dilatant for the majority of the *P*-*Q* space attainable in our apparatus. The dilatant failure envelopes for the andesites highlight that differential stress at failure increases linearly with effective mean stress, in accordance with the Mohr-Coulomb criterion. While it is common for porous sedimentary rocks to have parabolic compactive yield envelopes (Wong and Baud 2012), the andesitic lavas of this study have linear compactive envelopes. This is likely the result of the duality of the porosity (microcracks and pores), as previously suggested by Zhu et al. (2010). As mentioned above, an increase in confining pressure on the compactive side of the failure envelope reduces the differential stress required for the onset of shear-enhanced compaction. However, in a rock containing microcracks and pores, an increase in confining pressure must also close a larger proportion of the pre-existing microcracks. Therefore, for the same increase in confining pressure, the decrease in the differential stress required for C* may be less for a rock containing microcracks than for an initially microcrack-free rock. The result, in *P*-*Q* space, is a linear compactive envelope. We note that parabolic envelopes were observed for a porous trachyandesite from the Azores (Loaiza et al. 2012; Fig. 5a) and porous tuff (Zhu et al. 2011), both of which contain low initial microcrack densities.

We find, in general, that the amplitude of the failure envelope is lower when the porosity is higher. In other words, lava containing lower porosity is intact (or pre-failure) over a much larger stress space. This is best observed on our 3D plot where the differential stress at failure and the effective mean stress are plotted alongside the initial connected porosity (Fig. 5b). 3D yield caps are typically deployed in soil mechanics, but have also been successfully applied to rocks (see Cuss et al. 2003 and references therein). In these studies, the third axis is the porosity multiplied by the grain size; in our diagram, we have chosen to use initial connected porosity as our third axis, since volcanic rocks cannot be described by a grain size and, while an average pore size could be utilised here, we highlight that the pore size distribution of our rocks varies tremendously (Heap et al. 2014b), raising doubt over the applicability of an average pore size.

Contrary to our expectation, the 3D failure envelopes show that the amplitude of the failure envelope for LAH4 (porosity = 25 %) is larger than that of C8 (porosity = 18 %) (Fig. 5b). The cause of this discrepancy is likely the result of the difference in pore size distribution and the size of the largest pore between the two andesites. While LAH4 contains a large number of small pores, and few large pores (the largest is about just over 1 mm), C8 contains a much wider pore size distribution, including pores almost 2 mm in diameter (Heap et al. 2014b). The stress intensity is higher at the tips of cracks emanating from larger pores (Sammis and Ashby 1986). A crack will propagate when a critical stress is reached; therefore, the larger the pore, the lower the applied differential stress required for crack propagation (see also Heap et al. 2014c). Therefore, pore size should also be considered important in controlling the mechanical behaviour and failure mode of volcanic rocks, just as grain size is important for sandstones (Wong and Baud 2012). Another noteworthy observation is that the failure envelope for the trachyandesite from the Açores (porosity = 18 %, Loaiza et al. 2012) has a much larger amplitude than that of the andesite from Volcán de Colima with a comparable porosity (C8, porosity = 17 %; Fig. 5a). While this difference could be explained by the differences in microcrack density and/or the pore size, we highlight a potential role for the presence of phenocrysts. The trachyandesite from the Açores is aphanitic (the crystals are microlites), while the andesites from Volcán de Colima are porphyritic (crystals are as large as a couple of mm). Phenocrysts in volcanic rocks often contain microcracks and other defects (plagioclase can be twinned for example) and could therefore affect their mechanical behaviour, although no firm conclusions can be drawn from the available data.

## Operative micromechanical processes

**Fig. 6:**
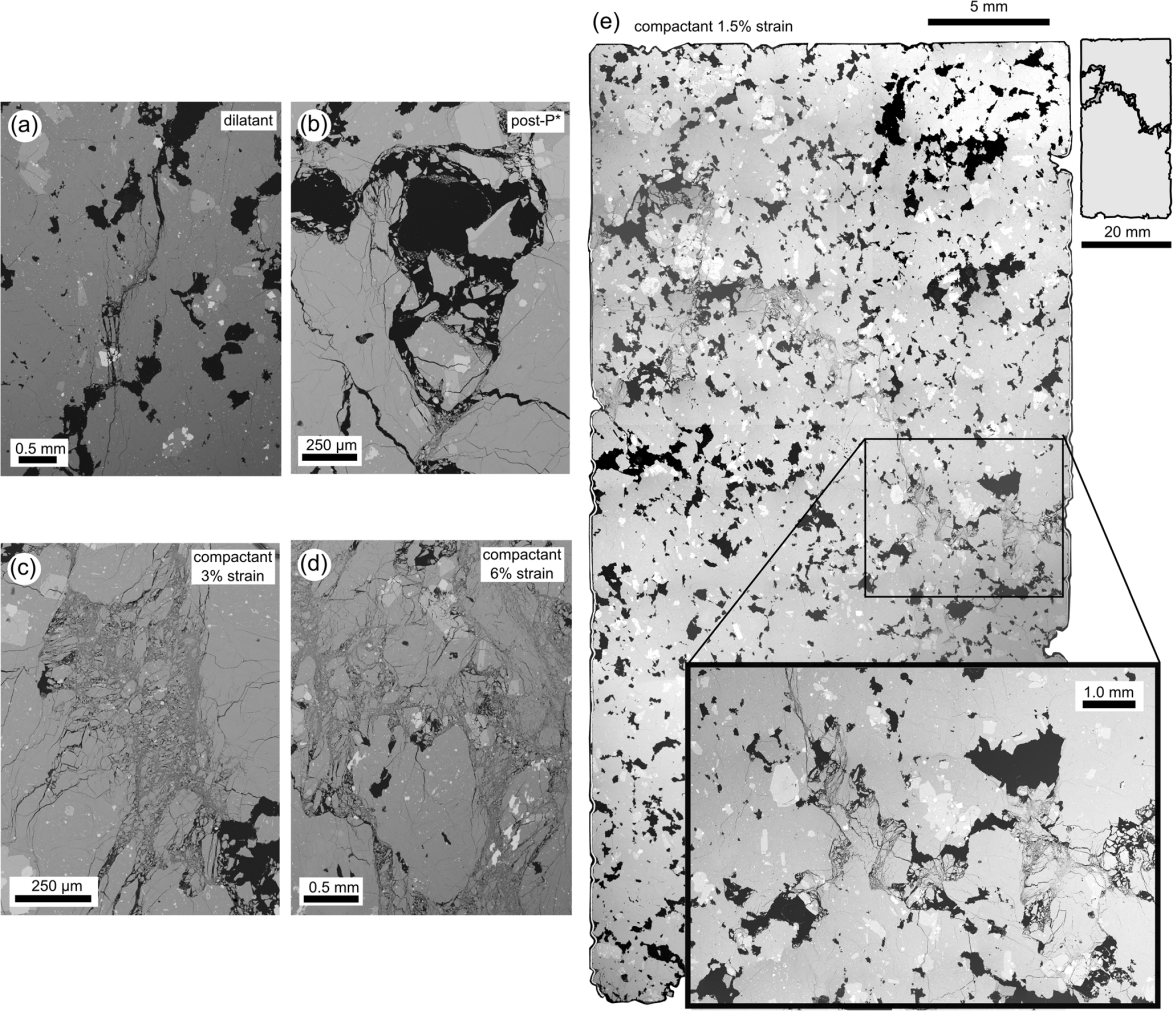
Microstructure. **a** Back-scattered scanning electron microscope (SEM) picture of pore-emanating microcracking from a dilatant constant strain rate experiment (Peff = 5 MPa) on a sample of C8. **b** SEM picture of cataclastic pore collapse during hydrostatic loading of a sample of C8 beyond the onset of hydrostatic pore collapse (P*). **c** SEM picture of cataclastic pore collapse from a compactant constant strain rate experiment (Peff = 50 MPa) on a sample of C8 taken to 3 % axial strain. **d** SEM picture of cataclastic pore collapse from a compactant constant strain rate experiment (Peff = 50 MPa) on a sample of C8 taken to 6 % axial strain. **e** SEM map showing a compaction localisation feature (band of collapsed pores) from a constant strain rate experiment (Peff = 50 MPa) on a sample of C8 taken to 1.5 % axial strain

It is well known that the formation of a shear fracture is the result of the nucleation, growth and coalescence of microcracks (e.g. Lockner et al. 1991). For porous materials, including rocks, microcracks usually emanate from pre-existing pores (e.g. Sammis and Ashby 1986; Wong and Baud 2012). Figure 6a shows a scanning electron microscope (SEM) image of a sample of andesite (C8) deformed within the dilatant regime (Peff = 5 MPa). We clearly see pore-emanated microcracks that are orientated sub-parallel to the maximum principal stress. We note that the microcracks shown in Fig. 6a form part of the macroscopic localised (i.e. the microstructure appears undisturbed outside the fracture) shear fracture.

Microstructurally, the inelastic compaction of porous rocks is typically attributed to cataclastic pore collapse and grain crushing (e.g. Wong and Baud 2012). Although microstructural observations have shown that pore collapse and grain crushing can be distributed throughout the sample (e.g. Menéndez et al. 1996), there are cases of compactive localisation. These features are well documented (in the field and laboratory) in porous sandstones (e.g. Mollema and Antonellini 1996; Baud et al. 2004) and limestones (e.g. Cilona et al. 2014), and are called compaction bands. Compaction bands in sandstones, for example, are subplanar surfaces of localised compaction—typically a few grains thick—orientated perpendicular to the maximum principal stress that show little or no evidence of shear. The porosity within the band is typically much lower than that of the surrounding host rock (e.g. Baud et al. 2006). During laboratory experiments, the appearance of compaction bands in sedimentary rock is typically associated with small stress drops (of a few MPa) and a sudden, temporary increase in the rate of AE activity (Baud et al. 2004, 2006).

Recently, two studies have shown evidence for compaction localisation in porous volcanic rocks (Loaiza et al. 2012; Adelinet et al. 2013). For example, Loaiza et al. (2012) showed that compaction localisation in porous trachyandesite deformed at a confining pressure of 130 MPa is manifest as bands of collapsed pores sub-perpendicular to the maximum principal stress. The structure was approximately 2 mm thick, roughly the average pore diameter. Small stress drops were seen in the stress-strain curves of these experiments, although AEs were not recorded during the experiments. The confining pressures required for the formation of compactive localisation in the trachyandesite were in excess of 95 MPa (i.e. at depths greater than about 4 km; Loaiza et al. 2012), perhaps too deep to be volcanologically relevant. The significance of these features within a volcano is that an experimental study on compactive localisation in sandstones has shown that permeability can be reduced by up to three orders of magnitude (Baud et al. 2012). As discussed above, the permeability of the country rock can impact sidewall outgassing, an important factor governing eruption explosivity (this is discussed further in the “Impact of failure mode on permeability” section).

To investigate the microstructural progression of our andesite lavas during compactive deformation, and to look for evidence of compaction localisation (as suggested by our mechanical data: we also observe the small stress drops associated with an increase in the rate of AE activity documented by Baud et al. 2004 and Baud et al. 2006), we performed three additional constant strain rate experiments on samples of C8 at an effective pressure of 50 MPa (corresponding to a depth of about 2 km) to axial strains of 1.5, 3 and 6 % (Table 1). As before (Fig. 3b), the stress-strain curves were punctuated by small stress drops associated with bursts of AE activity. We also performed an additional hydrostatic experiment to study the microstructure of a sample deformed beyond P* (Table 1). Similarly to previous studies on porous sedimentary rocks (Wong and Baud 2012) and volcanic rocks (Zhu et al. 2011; Loaiza et al. 2012), the acceleration in porosity reduction at P* seen here is the result of distributed pore collapse (Fig. 6b). Collapsed pores are partially filled with broken fragments of groundmass and are often bounded by microcracks (Fig. 6b).

An SEM map of the sample deformed to an axial strain of 1.5 %, i.e. immediately following the first stress drop, shows clear evidence of a compactive strain localisation feature (Fig. 6e). The feature, a band of collapsed pores (that have been infilled or partially filled with broken fragments of groundmass; see inset in Fig. 6e), traverses the diameter of the sample (20 mm) and is the thickness of the collapsed pore through which it passes (typically 0.25–0.5 mm). The band is not perpendicular to the maximum principal stress but is guided through the sample by the distribution of pores. Neighbouring collapsed pores are often connected by microcracks. We note that the pores appear undisturbed outside the band (i.e. the deformation is localised at the millimetre scale). Substantial pore collapse is seen in the samples deformed to 3 and 6 % strain (Fig. 6c, d). Due to the extent of the pore collapse, it is difficult to distinguish discrete bands of compacted pores. The observed deformation is likely the result of the amalgamation of several bands. We highlight that these cataclastic microstructures share similarities with the volcanic breccia found within the conduit zone of Unzen volcano, Japan (Goto et al. 2008).

Since a band is assumed to grow during a discrete stress drop and AE pulse (e.g. Baud et al. 2004), we can estimate (assuming uniaxial strain and that the bands are perpendicular to the maximum principal stress) that the inelastic axial strain associated with band growth is typically between 0.04 and 0.06 % for both C8 and LAH4 (corresponding to an axial shortening of about 20 μm). Microstructural observations indicate that the localised band has a thickness equal to the collapsed pore through which it passes (typically 0.25–0.5 mm), suggesting that the porosity reduction within the band is on the order of 4 to 8 %. In other words, the porosity is 17 % outside the band and about 10 % within the band. By contrast, the porosity of compaction bands in Bentheim sandstone was estimated to be about 8 %, considerably lower than the initial porosity of 23 % (Baud et al. 2004). These results are discussed further in the section “Impact of failure mode on permeability”. The ubiquity of cataclastic pore collapse during the deformation of porous volcanic rocks at high confining pressures (Zhu et al. 2011; Loaiza et al. 2012; Adelinet et al. 2013; Heap et al. 2014a, 2015) highlights the universality of pore collapse as the operative micromechanical mechanism driving low-temperature (below Tg) compactant deformation in porous volcanic rocks.

Field, experimental and modelling evidence suggest that the development of compaction bands is enhanced in well-sorted sandstones (Wang et al. 2008; Cheung et al. 2012). When the grain size distribution is large, compaction bands do not form because the deformation is accommodated by the smaller grains (Cheung et al. 2012). However, extrusive volcanic rocks cannot be characterised by a grain size. Nevertheless, in a similar manner, could compactive localisation features only occur in volcanic rocks with a homogeneous pore size distribution? It follows that, if the pore size distribution is wide, the deformation may focus on the larger pores (e.g. Heap et al. 2014c), resulting in distributed cataclastic pore collapse (assuming that the large pores are distributed throughout the sample). Compactive localisation features may therefore develop more easily when the pore size is relatively uniform. The collapse of one pore encourages the collapse of a neighbouring pore, due to the redistribution of stresses, promoting cascading pore collapse across the sample (in a similar way to cascading grain failure in the development of compaction bands in sandstones; Wang et al. 2008). However, we have observed compaction localisation in andesites with an extremely wide pore size distribution (C8; see Heap et al. 2014b; Fig. 6b). Firm conclusions on the favourable rock attributes for compaction localisation in volcanic rocks cannot be provided with currently available data, although we highlight a potentially important role for pore shape, a factor that displays much more variability in volcanic rocks than in sedimentary rocks. In a simplistic scenario where the pore shape is spherical, stresses are likely to focus on the larger pores, allowing the damage to be distributed throughout the sample. However, non-spherical pores may focus the deformation away from the larger pores and permit the formation of compactive localisation features through networks of misshapen pores. This interpretation is supported by the presence of large intact pores in C8 deformed to 1.5 % strain (Fig. 6).

## Impact of failure mode on permeability

Our experimental data demonstrate that edifice-building lavas can either dilate or compact in response to stress, depending on their depth and porosity. To explore permeability evolution as a consequence of dilatant and compactant failure modes, we measured the change in permeability of samples of porous (17 %) andesite (block C8) deformed in both regimes. Two samples (20 mm in diameter and about 20 mm in length) were loaded diametrically in uniaxial compression (at a constant strain rate of 10^−5^ s^−1^) until tensile failure, and two samples (20 mm in diameter and about 40 mm in length) deformed triaxially at a pore pressure of 10 MPa, a confining pressure of 60 MPa and a constant strain rate of 10^−5^ s^−1^ to axial strains of 1.5 and 4.5 %, respectively (Table 1). Gas (nitrogen) permeability was measured before and after deformation at a constant confining pressure of 1 MPa. We found that a tensile fracture—parallel to the imposed flow direction—serves to increase permeability by about a factor of two (permeability increased from 6.2 × 10^−13^ and 1.6 × 10^−12^ m^2^ to 1.0 × 10^−12^ and 2.8 × 10^−12^ m^2^ for the two samples, respectively). We note that (1) this increase may be reduced at confining pressures higher than 1 MPa (see Nara et al. 2011) and (2) a larger increase may be seen in andesites containing a lower initial porosity. The permeability of the fractured samples can be considered as an equivalent permeability (i.e. equal to the contribution of both the fracture and the host rock). Fracture permeabilities were calculated, using a fracture aperture of ~0.25 mm (determined through microstructural observations), to be 3.0 × 10^−11^ and 9.8 × 10^−11^ m^2^ for the two samples, respectively.

Compaction to 1.5 % strain reduced permeability from 6.2 × 10^−12^ to 2.6 × 10^−12^ m^2^ (a decrease by a factor of about two), and compaction to 4.5 % strain reduced permeability from 3.3 × 10^−12^ to 3.1 × 10^−13^ m^2^ (a decrease by about an order of magnitude). We highlight that compaction bands in sandstones resulted in a dramatic reduction in sample permeability (by up to three orders of magnitude, Baud et al. 2012). Our data suggest that a single band of collapsed pores—orientated perpendicular to the imposed flow direction—does not significantly reduce permeability and that this may be a result of a combination of their tortuous nature (gaps may exist over the area of the band) and the fact that the estimated porosity reduction within the band (4–8 %) is less than that typically estimated for compaction bands in sandstones (~15 %; Baud et al. 2012).

Taken together, these data suggest that the failure mode of the host rock will play an important role in conduit outgassing and therefore in dictating eruption characteristics: a dilatant failure mode in the upper conduit (<1 km) will assist outgassing, and compaction in the deep edifice (>1 km) will hinder outgassing (this is discussed further in the “Volcanological significance” section).

## Switching failure modes at high strains and the limit of compaction

As previously stated, porosity exerts a crucial role on the failure mode of rock (e.g. Wong and Baud 2012). However, we have also shown that porosity can be severely reduced during compactant deformation (Fig. 2). It follows that, after a certain degree of compaction, the rock may contain a porosity low enough to react to an applied stress in a dilatant manner. In rock mechanics, this strain-dependent switch in mechanical behaviour is referred to as C*′ and has been observed in porous limestones (e.g. Baud et al. 2000) and sandstones (e.g. Schock et al. 1973; Baud et al. 2006). C*′ will also provide us with a measure of the limit of inelastic porosity loss in porous andesitic edifice rocks. Prior to this study, this phenomenon had never been observed in porous extrusive volcanic rocks.

**Fig. 7:**
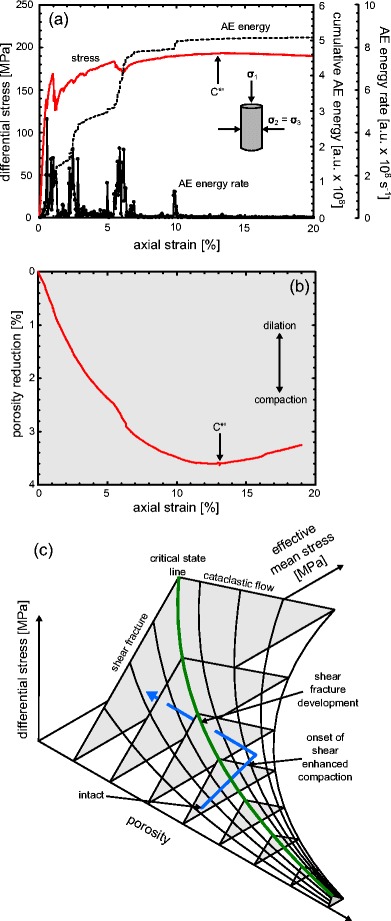
The strain-dependent switch to dilatant behaviour in porous andesite. **a** Stress-strain curve and the associated cumulative acoustic emission (AE) energy and AE energy rate (AE energy is given in arbitrary units, a.u.), for a constant strain rate experiment on a sample of C8 (Peff = 50 MPa) deformed to an axial strain of 20 %. **b** The porosity reduction with axial strain for the experiment shown in panel **a**. The position of the switch to dilatant behaviour C*′ is indicated on panels **a** and **b. c** 3D schematic diagram of differential stress, effective mean stress and initial connected porosity showing the path of a sample (*blue solid line*) deforming in the compactive regime to high strains. The sample eventually crosses the critical state line (the *green solid line*, the transition between compactant and dilatant behaviour) as a result of porosity reduction

To explore this concept in porous andesite, we performed a constant strain rate experiment on a sample of C8 at an effective pressure of 50 MPa to an axial strain of 20 % (Fig. 7). We find that the switch from compactant to dilatant behaviour, C*′, occurs at an axial strain of about 13 % and a porosity loss of about 3.6 % (for a sample containing an initial porosity of 15.5 %). In other words, for this sample, the maximum porosity loss as a result of inelastic compaction is 3.6 %, leaving the sample with a porosity of 11.9 %. Considerable porosity destruction may not therefore be obtainable in porous andesitic edifice rocks, although the porosity reduction at C*′ should increase for rocks containing higher initial porosities and at higher pressures (depths) (Baud et al. 2006).

**Fig. 8:**
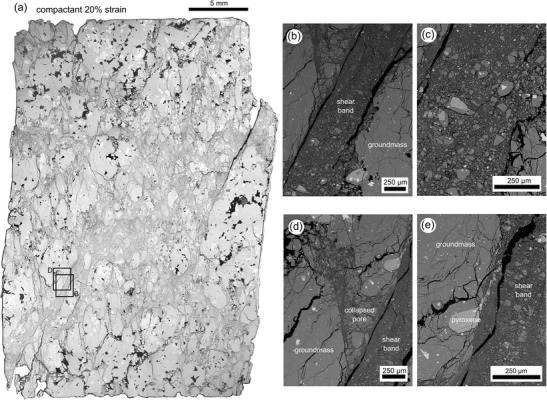
Microstructure. **a** Back-scattered scanning electron microscope (SEM) map of a sample of C8 deformed at a constant strain rate (Peff = 50 MPa) to an axial strain of 20 %. **b** SEM picture of one of the anastomosing shear bands. **c** SEM picture showing the crushed groundmass and crystals within the anastomosing shear band shown in panel **b. d** Crosscutting relationships. An anastomosing shear band overprinting a collapsed pore. **e** Crystal fragments entrained by the shear band and transported along the direction of shear

In a sample that has surpassed C*′, compactive pore collapse should be overprinted by a shear fracture. An SEM map of the deformed sample beyond C*′ is presented as Fig. 8a and shows a well-developed shear zone, up to 10 mm thick in places, of collapsed pores, intense fracturing and numerous anastomosing shear bands (Fig. 8b) containing fine-grained (from a few microns up to a few tens of microns) pulverised groundmass and crystals (Fig. 8c). Crystals on the boundary of the highly sheared bands have been fractured and the broken fragments have been transported parallel to the direction of shear (Fig. 8d). Outside the shear band, we notice that most of the pores are collapsed; the anastomosing shear bands often overprint evidence of cataclastic pore collapse (Fig. 8e). We again highlight the similarity between these microstructures and those of the volcanic breccia found within the conduit zone of Unzen volcano, Japan (Goto et al. 2008).

The switch in failure mode as porosity is reduced is best depicted on a 3D failure envelope (Fig. 7c). A theoretical “critical state line” can be mapped out schematically in *P*-*Q*-porosity space to delineate the transition between dilatant and compactant behaviour (see Fig. 7c); with progressive compaction (i.e. a reduction on the porosity axis), compactant volcanic materials will migrate towards this line. The switch in failure mode would be observed as the reduction in porosity allows the rock to cross the critical state line, as shown in Fig. 7c. We infer that highly strained rocks (in our experiment, C*′ required an axial strain of 13 %) near the conduit, or deep in the edifice, will be prone to this switch in failure mode (see “Volcanological significance” section). A strain-dependent switch to brittle failure has also been observed in high-temperature (940–945 °C) uniaxial deformation experiments on andesite from Volcán de Colima (Kendrick et al. 2013). However, in magma, the reduction in porosity required for a dilatant response is the consequence of viscous pore rearrangement and closure, rather than cataclastic pore collapse.

## Volcanological significance

**Fig. 9:**
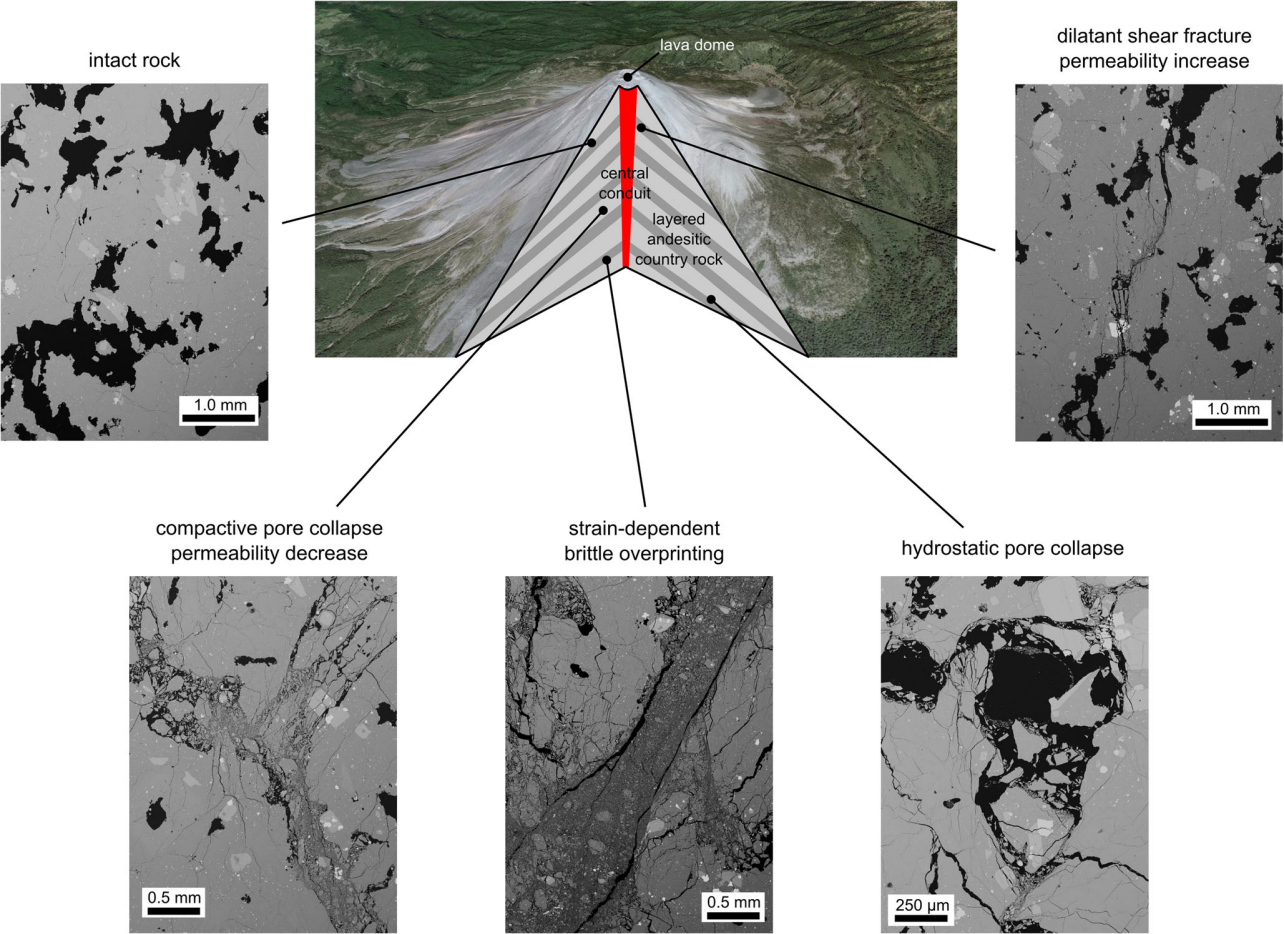
Schematic cross section of Volcán de Colima (layered andesitic edifice host rocks with a central conduit of dykes; image taken from Google Earth^TM^) . The cross section is annotated with back-scattered scanning electron microscope pictures of the intact material and the various deformation microstructures. See text for details

Our experimental data help constrain the depth of the transition between a dilatant and compactant failure mode in edifice-forming andesitic lavas. Based on these data, we have constructed a schematic cross section of Volcán de Colima that highlights regions of the volcano that are likely to (1) be intact (any deformation is elastic), (2) fail in a dilatant manner, (3) fail in a compactant manner or, (4) fail via inelastic lithostatic compaction (Fig. 9). We anticipate that differential stress will be higher closer to the central conduit of dykes and that effective pressure will increase with depth. Porous andesites will react to regional and local stresses in a dilatant manner in the shallow edifice (<1 km) and in a compactant manner at depths greater than about 1 km. It is worthwhile noting that the depth of the transition between a dilatant and compactant failure mode is likely reduced for rocks containing higher porosities and increased for rocks containing lower porosities. The strain-dependent switch to dilatant behaviour (C*′) is likely to be encountered deeper in the edifice, where older rocks have suffered significant inelastic strain. Inelastic lithostatic compaction (P*) can occur far from the sources of deformation but requires depths of at least 4–5 km (although we note that very porous rocks—such as pumiceous or scoracious rocks (see Farquharson et al. 2015)—may encounter inelastic lithostatic compaction at volcanologically relevant depths).

### Implications for lateral outgassing

The ease with which exsolved gases can escape the conduit can impact the style and intensity of an eruption; generally speaking, efficient outgassing promotes effusive behaviour whereas the retention of gas pressure promotes explosive behaviour (e.g. Eichelberger et al. 1986; Woods and Koyaguchi 1994; Rust et al. 2004; Mueller et al. 2008; Nguyen et al. 2014; Castro et al. 2014; Okumura and Sasaki 2014; Gaunt et al. 2014). The permeability of the edifice host lavas is likely to play an important role in the outgassing of the conduit magma (Jaupart 1998; Collombet 2009; Collinson and Neuberg 2012; Heap et al. 2014b; Farquharson et al. 2015); therefore, high-permeability host rocks may encourage effusive behaviour, and vice versa.

Our experimental data show that a throughgoing tensile fracture can increase sample permeability by a factor of two. Therefore, the dilatant deformation of edifice host rocks in the upper edifice (Fig. 9) may serve to increase permeability and assist the lateral outgassing of the conduit. However, we note that this increase in permeability may be suppressed at pressures high enough to close fluid pathways (e.g. Nara et al. 2011). Recent field evidence has exposed the ubiquitous presence of fractures within the dome, near the dome and on the upper flanks of Volcán de Colima (Kolzenburg et al. 2012; James and Varley 2012; Lavallée et al. 2015). Their presence, anticipated throughout the upper edifice (e.g. Heiken et al. 1988), serves as a testament to the ongoing brittle deformation and outgassing of the shallow edifice. Dilatant failure near the central conduit (the volume inferred to experience higher stresses) may create a permeable halo around the conduit down to a depth of about 1.5 km (i.e. the depth of the dilatant to compactant transition) that provides an efficient outgassing channel (e.g. Rust et al. 2004; Lavallée et al. 2013; Young and Gottsmann 2015). Further, outgassing through large-scale fractures and faults in the edifice is also supported by detailed field studies (e.g. Varley and Taran 2003).

Although edifice rocks are rarely above the temperature of their melt phase, preventing the efficient viscous sintering of fractures, we highlight that hot pressing (e.g. Kolzenburg et al. 2012) and mineral precipitation (e.g. Taran et al. 2001; Horwell et al. 2013; Schipper et al. 2015) may promote fracture sealing and permeability reduction between periods of unrest activity.

By contrast, rock will deform in a compactant manner deeper in the edifice (Fig. 9). Data from this study show that compaction can decrease permeability significantly (by an order of magnitude at a strain of 4.5 %). Therefore, the compactant deformation of deep edifice host rocks will serve to decrease permeability and impede the lateral outgassing of exsolving magma through the deep conduit wallrock. Evidence for persistent volcano subsidence at Volcán de Colima is provided by both in situ (Murray and Wooller 2002) and passive (Pinel et al. 2011) ground deformation methods. Subsidence rates as high as 93 mm per year (between 1982 and 1999) have been recorded at the edge of the dome, and based on the lack of consistency in horizontal movements, this subsidence has been interpreted as due to the compaction and settling of the edifice (Murray and Wooller 2002). If the ongoing compaction of Volcán de Colima is the result of compactive deformation, as presented herein, it implies ongoing reduction in the permeability of deep-edifice rocks (> 1 km). If faulting within the upper edifice cannot compensate for the continued compaction and permeability reduction of the rocks deeper in the edifice, the potential for explosivity at Volcán de Colima may be subject to increase over time.

Based on our data, we suggest that models of conduit outgassing (e.g. Collombet 2009; Collinson and Neuberg 2012) may be improved by considering permeability of the lower edifice (>1 km) to be lower than that of the upper edifice (<1 km).

### Implications for volcano stability

Fracturing in the upper edifice, as evidenced by the ubiquitous presence of fractures, is likely to reduce the integrity and structural stability of the edifice, leading to an increased risk of flank or large-scale dome collapse. Fault movement can result in bulging, intense fracturing and landsliding within the flanks, greatly destabilising the volcano (Lagmay et al. 2000). Subsequent intrusions of magma preferentially infiltrate heavily faulted domains of the volcano resulting in additional instability (Voight et al. 1983; Lagmay et al. 2000; Donnadieu and Merle 1998). However, we highlight that fracture-induced instability may be offset by the healing of fractures (e.g. Kolzenburg et al. 2012).

Although Volcán de Colima is characterised by persistent edifice subsidence (Murray and Wooller 2002; Pinel et al. 2011), interpreted as due to the compaction and settling of the edifice (Murray and Wooller 2002). There is no clear evidence of volcano spreading at Volcán de Colima (Murray and Wooller 2002), a key contributor to volcano instability (e.g. McGuire 1996; van Wyk de Vries and Francis 1997; Borgia et al. 2000). The lack of definitive evidence for volcano spreading may be explained by the relatively young age of Volcán de Colima (about 4000 years old; Murray and Wooller 2002). Volcanic spreading is one of the final stages of the development of a volcanic structure, preceded by periods of building, compressing, thrusting and intruding (Borgia 1994). Inelastic compaction of the edifice rocks may therefore be one of the principal mechanisms driving the “compressing” stage of the growth of a stratovolcano, representing an early stage in the growth and destruction cycles that have dominated the history of the Colima volcanic complex (Stoopes and Sheridan 1992; Cortés et al. 2010). Volcano growth and destruction cycles at the Colima volcanic complex are exemplified by the fact that Volcán de Colima is constructed within the amphitheatre of an earlier collapse structure (Fig. 1a). We speculate that, later in the life cycle of the volcano, the inelastic compaction of edifice-forming rock may also greatly assist volcano spreading and destabilisation. The substantial volume and distribution of previous collapses (Stoopes and Sheridan 1992 and references therein) highlight the extreme danger posed by Volcán de Colima.

## Concluding remarks and perspectives

The failure mode of edifice-forming lava depends on the physical attributes of the lava, primarily the porosity and the pore size, and the conditions under which it deforms. At shallow depths (<1 km), both low- and high-porosity lavas dilate and fail by shear fracturing. However, as depth increases, while low-porosity (<10 %) lava remains dilatant, the failure of high-porosity lava is compactant and driven, on the microscale, by cataclastic pore collapse. Importantly, the choice of failure mode dictates the evolution of key physical properties, such as permeability. Our study has shown that a throughgoing tensile fracture in a sample of porous andesite increases sample permeability by about factor of two and that inelastic compaction can reduce sample permeability by an order of magnitude. The outgassing of volatiles from the conduit may therefore be efficient in the shallow edifice, where rock can fracture, and impeded deeper in the edifice due to compaction. The failure mode of volcanic host rock, and the attendant implications for sidewall outgassing, is thus likely to influence the dominant eruption style: effusive or explosive. If faulting within the shallow edifice cannot compensate for the progressive compaction and permeability reduction of the rocks deeper in the edifice, the explosive potential of a volcano may be subject to increase over time. In terms of volcano stability, fracturing in the upper edifice—which can result in bulging, intense fracturing and landsliding within the flanks—is likely to reduce the integrity of the edifice and lead to an increased risk of flank or large-scale dome collapse. Deeper in the edifice, compactive deformation could explain volcano subsidence and assist in volcano spreading and destabilisation. We highlight that the implications of this study are by no means restricted to Volcán de Colima; due to the comparable construction and eruption histories, and porosity ranges of the edifice host rocks, these implications are likely relevant to similar active andesitic stratovolcanoes, such as Merapi (Indonesia), Santa María (Guatemala), Tungurahua (Ecuador) and Ruapehu (New Zealand).
